# Walking Training Enhances Corticospinal Excitability in Progressive Multiple Sclerosis—A Pilot Study

**DOI:** 10.3389/fneur.2020.00422

**Published:** 2020-06-04

**Authors:** Arthur R. Chaves, Augustine J. Devasahayam, Morten Riemenschneider, Ryan W. Pretty, Michelle Ploughman

**Affiliations:** ^1^Recovery and Performance Laboratory, Faculty of Medicine, L. A. Miller Centre, Memorial University of Newfoundland, St. John's, NL, Canada; ^2^Section for Sports Science, Department of Public Health, Aarhus University, Aarhus, Denmark

**Keywords:** transcranial magnetic stimulation, neuroplasticity, rehabilitation, exercise, progressive multiple sclerosis, corticospinal excitability, fatigue

## Abstract

**Background:** Inflammatory lesions and neurodegeneration lead to motor, cognitive, and sensory impairments in people with multiple sclerosis (MS). Accumulation of disability is at least partially due to diminished capacity for neuroplasticity within the central nervous system. Aerobic exercise is a potentially important intervention to enhance neuroplasticity since it causes upregulation of neurotrophins and enhances corticospinal excitability, which can be probed using single-pulse transcranial magnetic stimulation (TMS). Whether people with progressive MS who have accumulated substantial disability could benefit from walking rehabilitative training to enhance neuroplasticity is not known.

**Objective:** We aimed to determine whether 10 weeks of task-specific walking training would affect corticospinal excitability over time (pre, post, and 3-month follow-up) among people with progressive MS who required walking aids.

**Results:** Eight people with progressive MS (seven female; 29–74 years old) with an Expanded Disability Status Scale of 6–6.5 underwent harness-supported treadmill walking training in a temperature controlled room at 16°C (10 weeks; three times/week; 40 min at 40–65% heart rate reserve). After training, there was significantly higher corticospinal excitability in both brain hemispheres, reductions in TMS active motor thresholds, and increases in motor-evoked potential amplitudes and slope of the recruitment curve (REC). Decreased intracortical inhibition (shorter cortical silent period) after training was noted in the hemisphere corresponding to the stronger hand only. These effects were not sustained at follow-up. There was a significant relationship between increases in corticospinal excitability (REC, area under the curve) in the hemisphere corresponding to the stronger hand and lessening of both intensity and impact of fatigue on activities of daily living (Fatigue Severity Scale and Modified Fatigue Impact Scale, respectively).

**Conclusion:** Our pilot results support that vigorous treadmill training can potentially improve neuroplastic potential and mitigate symptoms of the disease even among people who have accumulated substantial disability due to MS.

## Introduction

Multiple sclerosis (MS) is a chronic neurodegenerative disease that causes structural (i.e., brain lesions and atrophy) and functional (i.e., neuronal connectivity and conduction alterations) central nervous system dysfunction (1). Most people with MS are initially diagnosed with the relapsing–remitting form of the disease (RRMS). RRMS is considered to be the inflammatory phase of MS with unpredictable development of central nervous system lesions that result in physical, sensory, and/or cognitive symptoms (i.e., relapses) (2). About 80% of people diagnosed with RRMS will eventually develop secondary progressive MS (SPMS), which is considered to be less inflammatory and more neurodegenerative (2, 3). As well, ~10% of people with MS present with primary progressive MS (PPMS), in which there is a steady disease progression from initial diagnosis of MS (2, 3). Several lines of evidence suggest that accumulation of disability in progressive MS is related to diminished capacity for neuroplasticity (2–4). Because most disease-modifying drugs act by reducing neuroinflammation, these same treatments do not seem to be as effective during progressive stages (5). Treatments that provide neuroprotection and enhancement of neuroplasticity to recover function and halt MS progression are highly warranted (6–10).

Animal and human research has shown that exercise enhances neuroplasticity by upregulating neurotrophins that facilitate cerebral gliogenesis, neurogenesis, synaptogenesis, and angiogenesis [for reviews see (11, 12)]. In some neurological conditions, such as Alzheimer's disease (13), stroke (12, 14), and spinal cord injury (15), exercise has also been shown to promote neuroplasticity. In MS, studies have shown that engagement in physical exercise training improves aerobic capacity (16, 17), physical function (e.g., walking capacity) (18), and mitigates physical symptoms (e.g., reduce fatigue, muscle weakness) (17, 19, 20). Recent studies support that a high degree of task practice (e.g., constraint-induced movement therapy) can enhance neuroplasticity in people with progressive MS (21), suggesting that there is continued capacity for plasticity even in later stages of the disease.

In humans, rehabilitation-induced neuroplasticity is typically measured using functional brain imaging (22, 23) and transcranial magnetic stimulation (TMS) (24). TMS generates a brief magnetic field through an insulated coil placed on the participant's scalp that induces neuronal activation of the primary motor cortex resulting in a motor-evoked potential (MEP) traveling through the corticospinal tract (24). Studies using TMS in healthy individuals have shown that exercise training promotes corticospinal excitability changes that are related to enhanced neuroplasticity (25–28). Typical TMS biomarkers that demonstrate exercise training-induced changes in corticospinal excitability include lower motor thresholds (29) and higher input-to-output MEP amplitudes responses (28), which are biomarkers mediated by increased glutamatergic (excitatory) neurotransmission (30). As well, in healthy individuals, exercise training has shown to reduce cortical silent period (CSP) duration (27, 31), an interruption of the electromyographic activity of a sustained muscle contraction after TMS-elicited MEP, suggestive of less activity of the inhibitory neurotransmitter gamma-aminobutyric acid (GABA) (24, 32).

Excessive GABAergic-mediated intracortical inhibition and lower corticospinal excitability measured with longer CSP and higher motor thresholds and lower input-to-output MEP amplitudes, respectively, are biomarkers of neurological impairment (e.g., stroke and MS) (10, 33–38) and reduced neuroplastic potential (39, 40). In MS, demyelination causes delay of the onset latency of the TMS-elicited MEP (41). Since MEP latency shortening is associated with recovery of physical function after stroke (42) and is faster in physically active individuals (29), in addition to excitatory and inhibitory TMS variables, MEP latency could also be altered by exercise (43). Although evidence from cross-sectional studies suggest a possible link between greater physical fitness and enhanced neuroplasticity in MS (44), no study has investigated the long-term effects of exercise training on neuroplasticity-like mechanisms using TMS, particularly in progressive stages of MS.

The primary aim of the present study was to investigate whether a rehabilitative walking training program induced corticospinal excitability changes related to enhanced neuroplasticity in people with progressive MS with severe MS-related walking disabilities. Since excessive fatigue is among the most disabling symptoms in progressive MS (18) and previous research has demonstrated the link between corticospinal excitability, fatigue (44–46), and fitness levels (44, 47), our secondary aim was to investigate whether exercise training-induced corticospinal excitability changes were associated with changes in physical fitness (cardiorespiratory fitness, body fat) (48) and subjective levels of fatigue (49, 50).

## Materials and Methods

### Experimental Design

This study was part of a feasibility and proof-of-principle interventional study aiming at restoring walking function among patients with MS-related walking disability (51). The data on feasibility and restoration of walking have been reported elsewhere (51). This interventional study (10 weeks, 3×/week exercise training) with TMS assessment pre, post, and 3-month follow-up was approved by the local health ethics board prior to initiation (Health Research Ethics Board, #2019.0225, NCT04066972).

### Participants

Ten participants were recruited via referral from neurologists and physiotherapists in the local MS clinic, as well as from an outpatient rehabilitation service discharge database. All participants signed informed consent prior to study inclusion. Recruitment and screening details have been described elsewhere (51). Participants were included if they (1) were diagnosed with progressive MS (SPMS or PPMS), (2) reported no relapses 3 months prior to inclusion, (3) presented with walking impairments (e.g., use of bilateral or unilateral gait aids), (4) had disability level ≥6.0 on the Expanded Disease Status Scale (EDSS), (5) were capable of participating in physical exercise [as per Physical Activity Readiness Questionnaire (PAR-Q) screening form (52)], and (6) were eligible to undergo TMS (53) and dual energy X-ray absorptiometry (DEXA) (54) as per screening procedures. Written informed consent was obtained from participants for the publication of any potentially identifiable images or data included in this article.

Two participants dropped out during the intervention (51), reporting not being able to commit to the proposed frequency of exercise sessions (3×/week). Eight participants (seven female) completed the intended exercise training, and pre–post data were collected. One participant (number 2) could not be reached during follow-up assessment. Participant demographics are presented in Table 1.

**Table 1:** Participants' demographics, body composition, and fitness.

**ID**	**MS Type**	**MS Severity (EDSS 0–10)**	**Walking Aid**	**Age Range (years)**	**DD (years)**	**Lean mass (Kg)**			**VO**_**2peak**_ **(mL.min^−1^${\text{kg}}_{\text{LM}}^{-1}$)**			**Body Fat %**		
						**Pre**	**Post**	**3-mo**	**Pre**	**Post**	**3-mo**	**Pre**	**Post**	**3-mo**
1	PPMS	6.5	Walker	55–60	10	57.22	58.47	59.88	20.05	21.71	19.48	45.6	46.6	46.5
2	SPMS	6.5	Walker	55–60	33	43.26	44.64	–	22.61	20.75	–	44.8	44.5	–
3	PPMS	6.5	Walker	40–45	19	54.99	57.06	57.63	24.79	34.28	29.74	35.1	35.4	34.6
4	SPMS	6.0	Cane	45–50	28	29.47	31.18	33.56	41.84	36.98	36.50	39.1	39.6	36.9
5	SPMS	6.5	Cane	35–40	19	54.31	56.05	54.52	33.31	37.87	41.17	39.1	40.0	37.8
6	SPMS	6.0	Cane	70–75	18	32.87	32.32	33.12	31.61	37.69	41.28	34.4	37.4	33.1
7	PPMS	6.5	Walker	70–75	10	–	–	–	27.31^#^	21.69^#^	18.09^#^	–	–	–
8	SPMS	6.0	Cane	25–30	2	41.74	43.56	42.62	48.28	48.66	48.13	44.7	40.8	39.9

*DD, disease duration; EDSS, Expanded Disability Status Scale; MS, Multiple Sclerosis; PPMS, primary progressive MS; SPMS, secondary progressive MS; 3-mo, 3-month follow-up*.

# *Participant 7 declined to undergo Dual Energy X-ray Absorptiometry, and the maximal (peak) volume of oxygen uptake (VO_2peak_ [mL.min^−1^${\text{Kg}}_{\text{LeanMass}\left(\text{LM}\right)}^{-1}$] was calculated by diving this participant's VO_2peak_ (mL.min^−1^) by the LM (kg) of total sample mean. 3-mo, 3-month follow-up*.

### Exercise Intervention

Participants underwent 10 weeks (3×/week) of vigorous treadmill walking exercise training in a temperature-controlled room (16°C) (51). The treadmill was equipped with a harness to prevent falls and to support ≤10% of participants' body weight. The dosage target of the exercise was 40 min (5 min warm-up and cool down) at a moderate-high intensity (40–65% heart rate reserve), which was adjusted throughout the training by increasing the speed and incline of the treadmill and/or reducing body weight support. Manual assistance to advance legs and resting breaks of ≤2 min were provided whenever necessary (51).

### Outcome Measures

All outcome measures were assessed before the intervention (*n* = 8), after the 10-week period intervention (*n* = 8) and at 3-month follow-up after the exercise intervention had ended (*n* = 7).

#### Cardiorespiratory Fitness

Levels of cardiorespiratory fitness were assessed as the peak rate of oxygen uptake (VO_2peak_ expressed in ml O_2_ min) during a graded maximal exercise test performed on a recumbent stepper (NuStep, Ann Arbor, MI, USA) as described elsewhere (14, 43, 44, 51, 55). Briefly, participants exercised at a cadence of 80 strides per minute while the equipment resistance level (1–10, beginning at level 3) was increased by one level every 2 min. If exhaustion was not reached at resistance level 10 (maximal NuStep resistance), the cadence was increased by 10 strides per minute every 2 min. Heart rate was continuously monitored during the test (H10, Polar Electro Inc., Kempele, Finland). The maximal and resting heart rate were used to calculate the proposed intensities of the exercise sessions [e.g., intensity target = 60% × (heart rate_Max_ – heart rate_Rest_) + heart rate_Rest_]. Fitness levels were calculated as the absolute VO_2peak_ (ml O_2_ min) relative to the total lean body mass (kg) (VO_2peak_ = ml O_2_ min^−1^ kg^−1^_leanmass_). The latter has been shown to be a more accurate measure of cardiorespiratory fitness in populations with a high body fat percentage (56).

#### Body Composition

Participants' total body weight (kg), body fat percentage (%), and lean body mass (kg) were assessed using whole-body dual energy X-ray absorptiometry (Discovery-A Densitometer, Hologic Inc., Bedford, MA, USA). Trained technicians calibrated the system prior to each assessment, and built-in software was used to analyze the data (v.12.6.1:3, Hologic Inc., Bedford, MA, USA).

#### Total Amount of Workload Performed During the Exercise Sessions

Total amount of workload performed was estimated using standardized equations (48). First, the VO_2_ (ml O_2_ min^−1^ kg^−1^) uptake during the exercise was calculated using the equation VO_2_ (ml O_2_ min^−1^ kg^−1^) = {resting component (3.5 ml O_2_ min^−1^ kg^−1^) + horizontal component [speed (m/min) ×0.1 ml O_2_ kg^−1^ m–^1^] + vertical component [1.8 ml O_2_ kg^−1^ m–^1^ × speed (m min^−1^) × incline_FractionalGrade_]}; adjustments for treadmill changes in speed and incline throughout the exercise were taken into consideration. The averaged VO_2_ (ml O_2_ min^−1^ kg^−1^) was transformed into metabolic equivalents. The kilocalorie (kcal)/minute was calculated using the equation kcal/min = (metabolic equivalents × 3.5 × total body weight in kg)/200. Finally, the total amount of workload performed was calculated by multiplying the kcal/minute by the total time in minutes that the participants exercised. These data were calculated from the first and the last exercise session participants performed during the exercise training and from the exercise session performed during the follow-up visit.

#### Levels of Fatigue

The intensity of fatigue perceived by the patients was assessed by the Fatigue Severity Scale (FSS) (49), whereas the impact of fatigue on activities of daily living was measured by the Modified Fatigue Impact Scale (MFIS) (50, 57) [for more details, see (51)].

#### Transcranial Magnetic Stimulation

Monophasic magnetic pulses were delivered to the right and left brain hemispheres using a BiStim 200^2^ stimulator (Magstim Co., Whitland, UK). With participants seated, a coil (70 mm figure-of-eight coil; Magstim Co. Whitland, UK) was positioned tangentially to the scalp with the handle pointing backwards and laterally at an 45° angle from the midline perpendicular to the central sulcus to deliver posterior–anterior directed pulses in the area of the primary motor cortex (58). Electromyographic (EMG) activity and MEPs were collected by surface electrodes (Kendall 200 Coviden, Mansfield, MA, USA) placed on the contralateral first dorsal interosseous hand muscle. Assessing corticospinal excitability on a non-exercised muscle (i.e., FDI rather than leg muscles) was considered important in order to more accurately investigate widespread effects on central nervous system mechanisms involved in brain plasticity (59, 60). A neuronavigation system (Brainsight, Rogue Research Inc., Montreal, QC, Canada) was used to ensure consistency of the coil position (i.e., angle and orientation) on participants' scalp during the TMS assessment. The Montreal Neurological Institute brain template was rendered in the BrainSight software and used as a 3-D stereotaxic template (61). The same system was used to collect EMG muscle activity and record MEPs with its built-in EMG system. The system collects at a sample rate of 3 kHz and uses a 2,500 V/V amplification and a gain of 600 V/V with a bandwidth of 16–550 Hz. Stronger and weaker hands were determined during baseline assessment (pre) by EMG recorded in the FDI muscle while participants performed a pinch grip maximal voluntary contraction (MVC) {mean EMG activity during MVC [stronger vs. weaker hand (mean ± SD)]: 106.07 ± 79.3 μV vs. 51.49 ± 45.12 μV; *Z* = −2.34, *p* = 0.018}. In order to be more precise when differentiating between stronger and weaker sides' brain-to-muscle connectivity (potentially less and more affected sides, respectively), EMG signal was prioritized over force production, since EMG represents the electrical activity from motor units firing action potentials generated by the central nervous system.

##### Motor thresholds and MEP latency

Suprathreshold TMS stimulations were delivered at different locations around the hand primary motor area. The location with the highest average peak-to-peak MEP amplitude was chosen as the hotspot. The hotspot was reassessed at pre, post, and follow-up, since it can show variability (62) and changes following interventions [e.g., exercise (63)]. The relative frequency method was used to determine resting motor thresholds (RMTs) and active motor thresholds (AMTs) (24, 64) and were determined as the minimum TMS intensity (maximal stimulator output percentage, MSO%) required to elicit peak-to-peak MEP amplitudes of ≥50 μV at rest (RMT) and ≥200 μV with participant performing 10% of pinch grip MVC (AMT) in at least 5 out of 10 trials. RMT and AMT are reported as MSO% (0–100). MEP latencies were determined from the valid MEPs collected during the RMT experiment and were calculated as the time [in milliseconds (ms)] between the TMS artifact and the MEP onset; the timepoint where the MEP amplitude surpassed ±2 standard deviation from the mean EMG background activity (100 ms prior to the TMS stimulation).

##### Excitatory and inhibitory recruitment curves

To create recruitment curves, TMS stimulation intensities of 105–155% of AMT (increments of 10%) were employed in randomized order with participants performing a pinch grip at 10% of MVC (47). Three to six stimulations (28, 65, 66) were delivered at each intensity, and the averaged peak-to-peak MEP amplitude (μV) and CSP time (ms) were recorded. CSP was defined as the time between the MEP onset to the return of EMG activity (≥±2 standard deviation from background EMG activity) (24). MEP amplitudes were normalized to the largest peak-to-peak amplitude (25) collected during baseline assessment (i.e., first TMS session; prior to beginning of the exercise training). A linear relationship between the normalized MEP amplitudes against the used TMS intensities (105–155% of AMT) determined the excitatory recruitment gain and accuracy (slope and *R*^2^ of the linear relationship, respectively) of the corticospinal tract in recruiting neurons (25, 34), both previously reported potential biomarkers of corticospinal tract integrity (67). Similarly, the inhibitory recruitment curve slope and *R*^2^ was calculated by plotting the CSP time against the TMS intensities. As an estimate of overall corticospinal excitation (MEP amplitudes) and inhibition (CSP time), the area under the curve was calculated using the trapezoid rule Δ*X* × (*Y*1 + *Y*2)/2, with *X* being the TMS intensity used (105–155% of AMT) and *Y* being the normalized MEP amplitudes (% of largest baseline MEP) or the recorded CSP time.

### Statistical Analysis

*A priori*, we planned to use a one-way repeated measures analysis of variance and Friedman test when testing normal and non-normally distributed data, respectively. Because tests of normality (e.g., Shapiro–Wilk) typically require samples sizes of *n* ≥ 10 to generate reliable results (68), the more robust non-parametric alternative (i.e., Friedman test) (69) was preferred (70) to determine changes in TMS variables [RMT, AMT, and excitatory and inhibitory recruitment curves (MEP amplitudes_105−155%AMT_, CSP time_105−155%AMT_, slope, *R*^2^, and area under the curve)], fitness (ml min^−1^ ${\text{kg}}_{\text{LM}}^{-1}$, body fat %), and workload performed (kcal/session), at the different time points (pre, post, and follow-up). Analysis between time points (pre vs. post vs. follow-up) is reported as $\chi_{\left(\text{degreesoffreedom}\right)}^{2}$ = test statistic, *p*-value. When statistically significant (*p* < 0.05), Bonferroni-corrected pairwise comparisons were performed to identify the difference across time points, and the adjusted *p*-value for multiple comparisons is reported. All data in the text are presented as median (Mdn).

Relationships between changes in cardiorespiratory fitness (ml min^−1^ kg^−1^_leanmass_), lean mass (kg), body fat (%), levels of fatigue (FSS, MFIS), workload performed (kcal/session), and TMS changes were investigated with Spearman's coefficient (rho) at the unadjusted significance level of *p* < 0.05. Change scores were calculated as % changes = post – pre/pre.

Differences between TMS values of the stronger and weaker hand were investigated separately for each time point (pre, post, follow-up) with Wilcoxon non-parametric paired *t*-tests.

## Results

### Exercise Training Increased Corticospinal Excitability in Both Hemispheres

**Figure 1:**
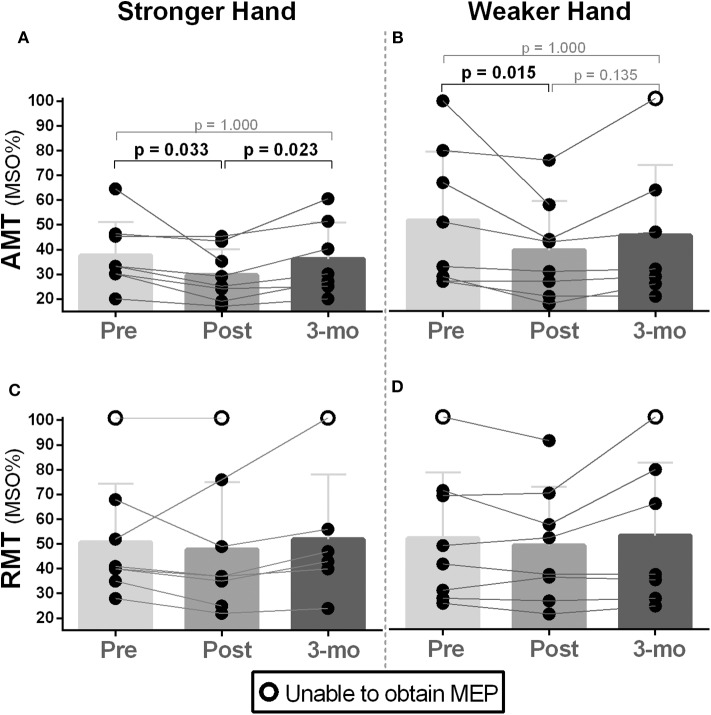
Effects of 10-week treadmill walking exercise training on active and resting motor thresholds. **(A,B)** Increased corticospinal excitability (CSE) was noted during active motor threshold (AMT) assessment in both brain hemispheres (i.e., corresponding to the weaker and stronger hands) as lower values of the maximal stimulator output (MSO%) were needed to elicit motor-evoked potentials (MEPs) in the contralateral first dorsal interosseous muscle (200 μV amplitude MEPs collected during 10% of pincer grip maximal voluntary contraction). AMT returned to baseline during the 3-month follow-up period assessment (3 mo). **(C,D)** There was no difference in MSO% between time points (pre, post, 3-month follow-up) for resting motor threshold (RMT) (i.e., MEPs collected during resting) measured in the hemisphere corresponding to the weaker hand. Because the absence of MEPs is an outcome that represents too low CSE (i.e., 100% of MSO not eliciting MEPs) (71), participants in this condition are represented as open circles. Preintervention, too low CSE (i.e., no MEPs) was noted in participant 2's stronger and weaker hands during RMT assessment. This participant's weaker hand demonstrated some recovery of CSE post-intervention as RMT's MEPs could be elicited at 92% of MSO. Lowered CSE (no MEPs) at 3-month follow-up was noted in participant 8's weaker hand as AMT and RMT could not be recorded.

Friedman's test showed a significant difference for AMT between time points (pre, post, follow-up) in both stronger and weaker hands [χ^2^_(2)_ ≥ 8.27, *p* ≤ 0.016]. Pairwise analysis revealed higher corticospinal excitability (i.e., lower AMT) in participants post- compared to pre-intervention in both stronger [MSO%; Mdn (pre vs. post) = 33 vs. 27, *p* = 0.033] and weaker hands [MSO%; Mdn (pre vs. post) = 41 vs. 37, *p* = 0.013), which returned to baseline at follow-up (Figures 1A,B). Higher variability was found for RMT; no change, increases, and decreases of RMT were noted across participants in both hemispheres (stronger and weaker hands), and no statistically significant changes were observed in either hemisphere (Figures 1C,D).

**Figure 2:**
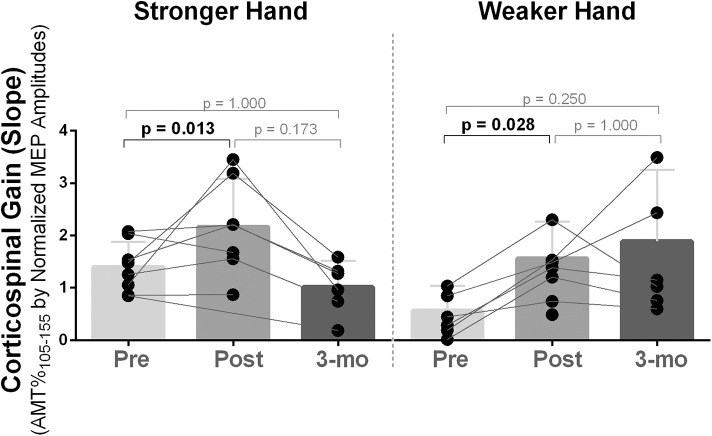
Effects of 10-week treadmill walking exercise training on corticospinal gain. After 10 weeks of exercise training, availability to recruit corticospinal tract neurons with increased transcranial magnetic stimulation intensities was increased (i.e., higher slope) in both brain hemispheres corresponding to the stronger and weaker hands and returned to baseline at 3-month follow-up (3-mo), although, two participants (numbers 6 and 8) continued to increase corticospinal gain in the hemisphere corresponding to the weaker hand during follow-up. The recruitment curve as collected using transcranial magnetic stimulation intensities of 105–155% of the active motor threshold (AMT) (increments of 10%) and the slope was determined from a linear regression between the normalized MEP amplitudes [% of the largest baseline motor-evoked potential (MEP)] against the TMS intensities performed (105–155% of AMT).

Corticospinal gain (excitatory recruitment curve slope) was statistically different between time points in both stronger and weaker hands [χ^2^_(2)_ ≥ 8.40, *p* ≤ 0.015]. Pairwise analysis revealed increased capacity to recruit excitatory neurons with increased TMS stimulation intensities (i.e., higher slope) post- compared to pre-intervention [Mdn = (pre vs. post) = stronger: 1.33 vs. 2.20, *p* = 0.013; weaker: 0.67 vs. 2.08, *p* = 0.028], which returned to baseline at follow-up (Figure 2). Recruitment curve accuracy (*R*^2^) did not change in neither stronger or weaker hand [χ^2^_(2)_ ≤ 4.00), *p* ≥ 0.135].

**Figure 3:**
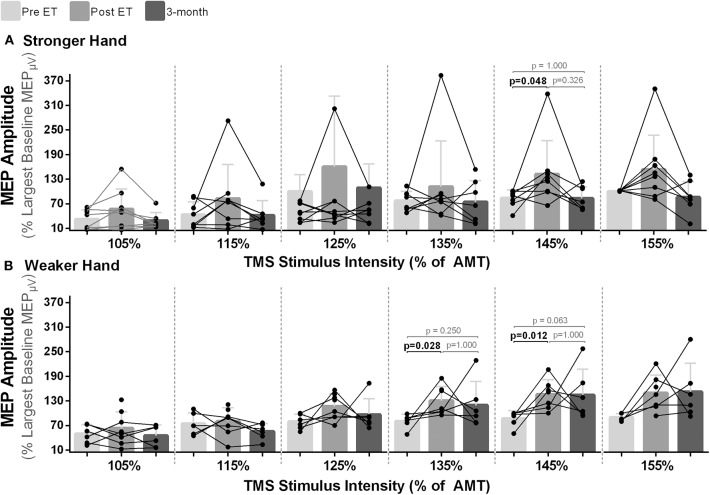
Effects of 10-week treadmill walking exercise training on motor-evoked potential (MEP) amplitudes. **(A)** Higher normalized MEP amplitudes (% of largest baseline MEP) demonstrate higher corticospinal excitability after the exercise training (ET) with return to baseline at 3-month follow-up (3-mo) in the hemisphere corresponding to the stronger hand at a transcranial magnetic stimulation (TMS) intensity of 145% of the active motor threshold (AMT) and **(B)** in the hemisphere corresponding to the weaker hand at the TMS intensities of 135 and 145% of the AMT.

For MEP amplitudes, statistical significance between time points were noted at the intensities of 135% [χ^2^_(2)_ = 7.00, *p* = 0.030] and 145% [χ^2^_(2)_ = 9.33, *p* = 0.009] of AMT in the weaker hand and at 145% of AMT in the stronger hand [χ^2^_(2)_ = 6.00, *p* = 0.050]. In all cases, pairwise analysis revealed increased corticospinal excitability (higher normalized MEP amplitudes) post- compared to pre-intervention with return to baseline at follow-up [% of largest baseline MEP; Mdn (pre vs. post): weaker hand: 135% of AMT: 85.49 vs. 111.39, *p* = 0.028; 145% of AMT: 85.78 vs. 151.66, *p* = 0.012; stronger hand: 145% of AMT: 88.73 vs. 127.05, *p* = 0.048; Figure 3].

### Exercise Training Reduced Intracortical Inhibition in the Hemisphere Corresponding to the Stronger Hand

**Figure 4:**
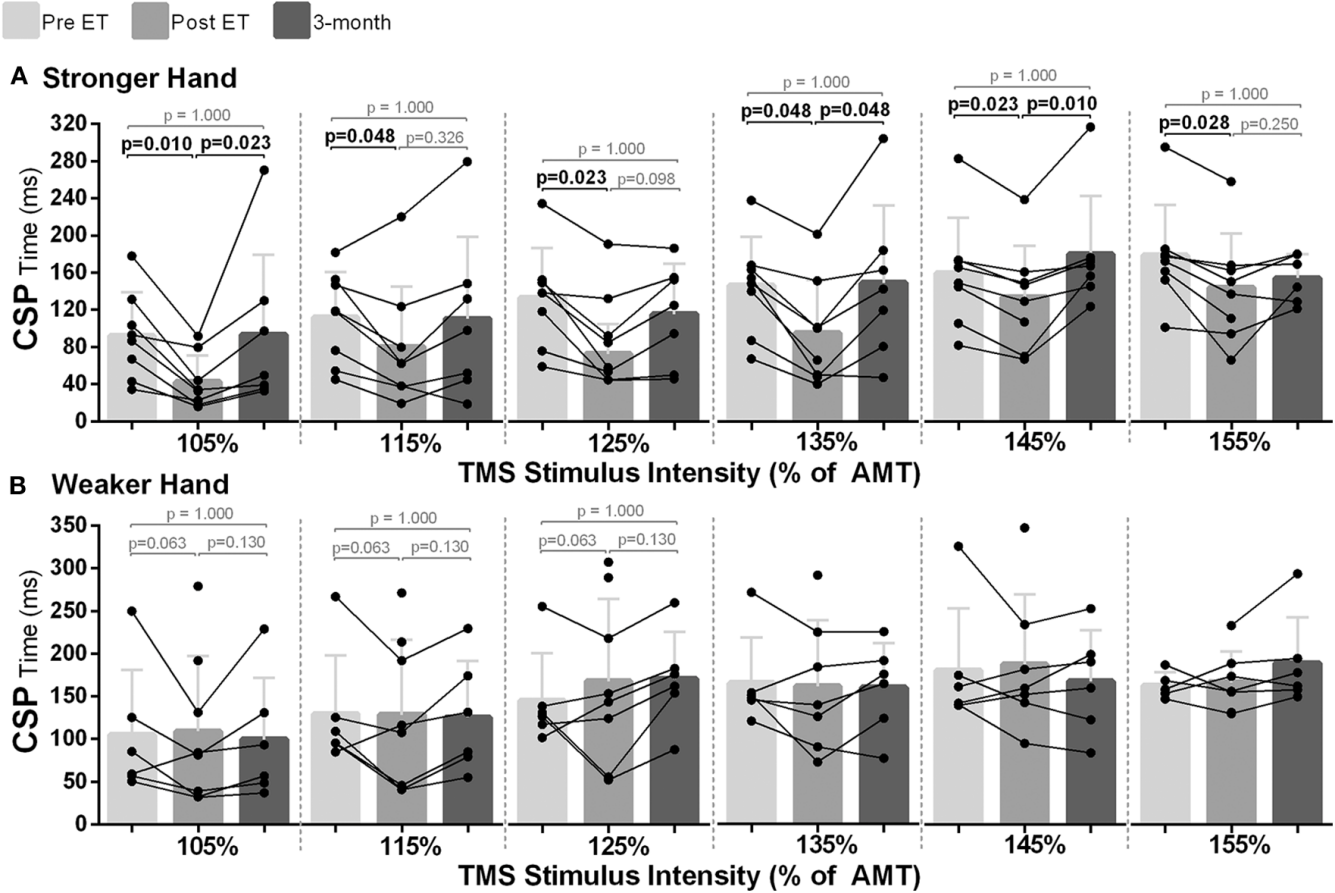
Effects of 10-week treadmill walking exercise training on cortical silent period (CSP) time. **(A)** In the hemisphere corresponding to the stronger hand, shorter CSP time (ms) at all transcranial magnetic stimulation intensities used [105–155% of active motor threshold (AMT)] suggested less GABAergic-mediated intracortical inhibition post-exercise training (ET), with return to baseline at 3-month follow-up (3-mo). **(B)** In the hemisphere corresponding to the weaker hand, although statistical significance was reached for the TMS intensities of 105, 115, and 125% of AMT between the different time points [Friedman's test: pre vs. post vs. 3-mo; χ^2^_(2)_ = 6.33, *p* = 0.042], there was no statistical significance during pairwise analysis.

In the stronger hand, differences between time points were noted for CSP investigated in all TMS intensities [105–155% of AMT; χ^2^_(2)_ ≥ 6.00, *p* < 0.050]. Pairwise analysis revealed reductions in CSP time post- compared to pre-intervention across all intensities used (*p* ≤ 0.048), which returned to baseline level at follow-up (Figure 4A). In the hemisphere corresponding to the weaker hand, there was a statistically significant difference for CSP time at the different time points at lower TMS intensities {105–125% of AMT [χ^2^_(2)_ = 6.33, *p* = 0.042]}; however, statistical significance was not reached during pairwise analysis (*p* ≥ 0.063; Figure 4B).

### Changes in Body Composition, Fitness, and Exercise Performance

**Figure 5:**
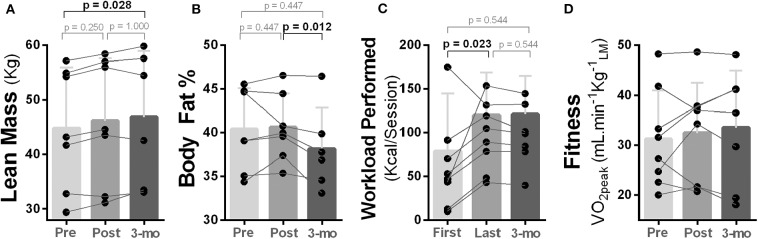
Effects of 10-week treadmill walking exercise training on body composition and physical fitness. **(A)** Amount of lean body mass (kg) measured using dual energy X-ray absorptiometry (DEXA) was higher at 3-month follow-up (3-mo) compared to pre-exercise training. **(B)** Body fat percentage (%) measured using DEXA was lower at 3-month follow-up compared to post-exercise training. **(C)** Participants were able to perform a higher exercise workload (kcal/session) at their last exercise session compared to the first. Total amount of workload performed was estimated using standardized equations (49). **(D)** No change was noted for cardiorespiratory fitness measured as peak rate of oxygen uptake during a graded maximal exercise test [VO_2peak_ = ml min^−1^ ${\text{kg}}_{\text{ofleanmass}\left(\text{LM}\right)}^{-1}$].

Lean body mass of the participants increased from pre- to post-intervention and from post-intervention to follow-up; however, only the change from pre to follow-up was statistically significant [χ^2^_(2)_ = 7.00, *p* = 0.030; Mdn, lean mass (kg) (pre vs. follow-up): 41.74 vs. 48.57, *p* = 0.028] (Figure 5A). Body fat also decreased during follow-up, and a statistical significance was noted from post to follow-up [χ^2^_(2)_ = 8.33, *p* = 0.016; Mdn, body fat % (post vs. follow-up): 40.00 vs. 37.35, *p* = 0.012; Figure 5B].

Although four out of eight participants improved their cardiorespiratory fitness (ml min^−1^ kg^−1^_leanmass_), no overall statistical change was reached (*p* ≥ 0.368; Figure 5D). However, an increased capacity to perform exercise were noted as participants were able to perform a higher exercise workload (kcal/session) in the last compared to the first exercise session [χ^2^_(2)_ = 7.14, *p* = 0.028; Mdn, kcal/session (pre vs. post) = 121.39 vs. 70.24, *p* = 0.023], and this capacity was maintained during follow-up (Figure 5C).

### Overall Corticospinal Excitation Increased Post-intervention in the Stronger Hand and Was Associated With Reductions in Fatigue

**Figure 6:**
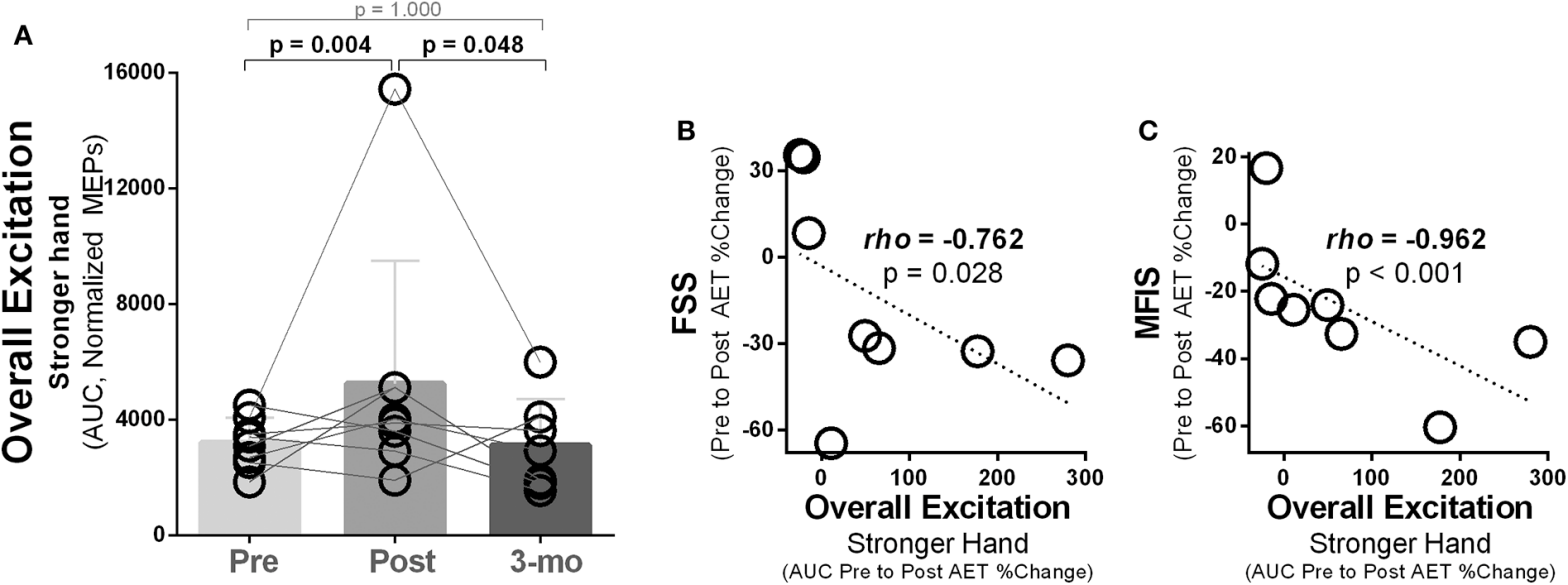
Ten weeks of treadmill walking exercise training induced increased overall corticospinal excitation that was associated with reductions in subjective fatigue. **(A)** In the hemisphere corresponding to the stronger hand, higher overall corticospinal excitation was noted post-exercise training, with complete return to baseline during 3-month follow-up (3-mo). Overall excitation was calculated as the area under the curve (AUC) using the trapezoid rule Δ*X* × (*Y*1 + *Y*2)/2, with *X* being the transcranial magnetic intensities used (105–155% of AMT; increments of 10%) and *Y* being the normalized motor-evoked potential (MEP) amplitudes (% of largest baseline MEP). **(B)** Increases in overall excitation (AUC) in the hemisphere corresponding to the stronger hand were associated to reductions in subjective levels of fatigue measured using the fatigue severity scale (FSS) and **(C)** the modified impact scale (MFIS).

In the stronger hand, overall corticospinal excitation [area under the curve (AUC), normalized MEP amplitudes] differed between time points [χ^2^_(2)_ = 11.14, *p* = 0.004]. Pairwise analysis revealed increased overall corticospinal excitation (higher AUC) post- compared to pre-intervention [Mdn, AUC_105−155%ofAMT_ (pre vs. post) = 3,237 vs. 3,947, *p* ≤ 0.016) with returned to baseline level at follow-up (Figure 6A). Relationship analysis demonstrated that greater increases in overall corticospinal excitation in the stronger hand were associated with greater reduction in fatigue severity levels measured with the FSS (rho = 0.762, *p* = 0.028; Figure 6B) and fatigue impact measured with the MFIS (rho = 0.962, *p* = 0.001; Figure 6C).

Nerve conduction speed (MEP latency) did not change in either side [χ^2^_(2)_ ≤ 1.14, *p* ≥ 0.565; Mdn, milliseconds (pre vs. post vs. follow-up): stronger hand, 24.17 vs. 24.51 vs. 22.12; weaker hand, 26.26 vs. 25.94 vs. 25.97].

All the TMS values (median and range), differences between stronger and weaker hands across time points, and reasons for missing values across time points are reported in Table 2.

**Table 2:** Transcranial magnetic stimulation values between stronger and weaker sides.

**Median (range)**	**Pre training**			**Post training**			**3-month follow up**		
**TMS variable**	**Stronger**	**Weaker**	**Sig**.	**Stronger**	**Weaker**	**Sig**.	**Stronger**	**Weaker**	**Sig**.
RMT (MSO%)	40 (28–68)^a^	45 (30–73)^a^	0.618	37 (22–76)	48 (26–92)	0.205	43 (24–56)^d^	40 (29–81)^f^	0.138
AMT (MSO%)	33 (20–64)	42 (27–100)	0.058	27 (17–45)	37 (18.76)	0.042^*^	30 (20–60)^e^	31 (21–64)^f^	0.307
MEP_105%AMT_	231.13 (186.67–331.17)	415.5 (181.5–464.25)^b^	0.046^*^	477.18 (243.50–1097.17)	222.50 (124.17–1072.20)	0.012	374.60 (91.50–634.40)^e^	295.88 (165.33–358.67)^f^	0.116
MEP_115%AMT_	310.00 (96.75–1398.00)	593.05 (174.00–1130.00)^b^	0.463	621.21 (319.00–1422.75)	320.75 (172.75–1720.80)	0.050	430.75 (146.25–1360.50)^e^	370.42 (153.00–860.33)^f^	0.600
MEP_125%AMT_	344.92 (199.50–2640.00)	818.47 (161.00–1365.77)^b^	0.753	740.50 (209.47–1592.00)	510–13 (226.60–3030.33)	0.779	597.40 (213.20–2100.00)^e^	772.17 (228.67–1453.75)^f^	0.753
MEP_135%AMT_	672.58 (248.00–3546.00)	550.23 (206.00–1483.67)^b^	0.345	1348.75 (353.33–1722.25)	665.50 (237.75–3587.33)^c^	0.237	724.20 (117.00–4664.40)^e^	994.67 (232.67–2159.67)^f^	0.463
MEP_145%AMT_	568.00 (334.50–3727.80)	564.63 (248.00–1812.67)^b^	0.345	1784.88 (430.33–4608.00)	765.67 (310.50–3998.00)^c^	0.128	1065.67 (272.00–4634.00)^e^	1165.08 (260.33–2814.80)^f^	0.345
MEP_155%AMT_	1037.55 (357.00–3771.33)	870.67 (468.50–1933.00)^b^	0.686	2047.85 (373.75–4031.20)	892.50 (232.33–4268.00)^c^	0.091	1346.17 (98.00–4669.00)^e^	1252.75 (257.20–2798.00)^f^	0.249
eREC slope (gain)	14.80 (3.53–77.38)	3.14 (−1.83–30.00)^b^	0.075	28.41 (3.22–82.06)	10.18 (1.70–66.77)^c^	0.091	15.51 (0.90–49.28)^e^	19.11 (2.24–59.03)^f^	0.686
eREC R^2^ (accuracy)	0.77 (0.51–0.97)	0.35 (0.00–0.97)^b^	0.173	0.76 (.042–0.96)	0.82 (0.66–0.99)^c^	0.499	0.78 (0.05–0.87)^e^	0.91 (0.82–0.95)^f^	0.043^*^
eREC AUC (overall excitation)	25852 (13182–126385)	30498 (7558–65129)^b^	0.463	58744.17 (16940.42–108246.00)	30189.83 (11258.50–150065.17)^c^	0.176	34050.50 (8432.00–154112.00)^e^	40965.42 (10859.33–83448.00)^f^	0.463
CSP_105%AMT_	89.56 (34.65–177.40)	72.47 (50.61–249.76)^b^	0.249	33.35 (16.17–91.63)	82.77 (31.92–279.04)	0.012^*^	49.66 (32.80–269.32)^e^	75.26 (37.17–229.93)^f^	0.249
CSP_115%AMT_	118.31 (45.06–181.49)	102.27 (84.86–266.75)^b^	0.345	62.34 (19.35–219.98)	112.16 (40.96–271.27)	0.036^*^	98.03 (18.88–279.07)^e^	108.28 (55.22–229.40)^f^	0.046^*^
CSP_125%AMT_	138.49 (58.90–233.98)	128.85 (101.84–255.44)^b^	0.116	71.41 (44.46–190.77)	148.58 (52.44–307.23)	0.017^*^	124.91 (45.60–186.06)^e^	169.13 (87.86–259.93)^f^	0.046^*^
CSP_135%AMT_	151.60 (67.87–237.88)	150.01 (121.46–272.03)^b^	0.345	83.57 (40.51–201.44)	140.24 (73.29–292.23)^c^	0.018^*^	142.76 (47.85–304.16)^e^	170.69 (77.56–225.96)^f^	0.173
CSP_145%AMT_	157.33 (82.26–282.32)	151.81 (139.38–325.93)^b^	0.173	137.96 (67.22–238.43)	159.61 (94.94–347.51)^c^	0.018^*^	167.32 (123.66–316.11)^e^	175.15 (83.65–252.69)^f^	0.345
CSP_155%AMT_	174.75 (101.28–294.92)	158.33 (146.92–187.09)^b^	0.893	143.82 (66.13–257.88)	156.08 (129.73–233.08)^c^	0.028^*^	156.71 (121.33–179.94)^e^	169.94 (149.51–293.69)^f^	0.046^*^
iREC slope (Gain)	1.88 (0.91–3.68)	1.84 (0.83–2.19)^b^	0.893	2.04 (0.89–2.56)	1.97 (0.29–2.88)^c^	0.866	2.17 (0.75–2.73)^e^	1.76 (1.03–2.27)^f^	0.345
iREC R^2^ (accuracy)	0.94 (0.75–0.99)	0.88 (0.84–0.99)^b^	0.893	0.88 (0.67–0.97)	0.87 (0.01–0.95)^c^	0.237	0.90 (0.73–0.93)^e^	0.70 (0.54–0.79)^f^	0.028^*^
iREC AUC (overall Inhibition)	6975.5 (3262.0–11718.0)	6531.5 (5823.00–12450.30)^b^	0.249	4369.13 (2271.20–10254.75)	6666.25 (3601.05–13043.85)^c^	0.018^*^	5857.20 (3397.85–7825.15)^e^	7482.20 (3976.30–12292.90)^f^	0.116
MEP latency (ms)	24.17 (21.38–43.15)^a^	26.26 (20.45–35.52)^a^	0.866	24.51 (19.48–43.78)	25.95 (20.36–38.02)	1.000	22.12 (21.88–29.69)^e^	25.97 (20.26–28.20)^f^	0.686

*AMT, active motor threshold; CSP, cortical silent period; eREC, excitatory recruitment curve; iREC, inhibitory recruitment curve; MEP, motor evoked potential; MSO%, maximal stimulator output percentage; RMT, resting motor threshold; eREC Slope = MEP Amplitude (μV) by TMS intensity_105−155%AMT_; iREC Slope = CSP time (ms) by TMS intensity_105−155%AMT_; Area under the curve (AUC) was calculated for both excitatory and inhibitory RECs using the trapezoid rule ΔX x (Y1+Y2)/2, whereby X were the MSO% used (i.e., X axis values, 105–155% of AMT) and Y are the recorded CSP lengths (ms) or the MEP amplitudes (μV)*.

* *Difference between stronger and weaker hand is statistically significant at α <0.05*.

a *Missing data from participant 2 due to too low corticospinal excitability (i.e., no resting MEPs)*.

b *Missing data from participant 2 and 7 due to too high AMT (AMT = 100 and 82%, respectively), thus the required increases in MSO% based on AMT to assess the REC could not be performed)*.

c *Missing data from participant 7 due to high AMT (AMT = 76%), thus the required intensities of 135–155% of AMT could not be performed, and the slope, R^2^ and AUC could not be calculated)*.

d *Time point with n = 5 (participant 2 could not be reached during follow-up assessment, missing data from participant 7 and 6 due to too low corticospinal excitability (i.e., no resting MEPs) and overheating of equipment (i.e., stimulator)*.

e *Missing data from participant 2 (could not be reached during follow-up)*.

f *Missing data from participant 2 (could not be reached during follow-up) and 7 [too low corticospinal excitability (i.e., no resting or contracting MEPs (RMT and AMT)]*.

## Discussion

We undertook this study to determine whether a 10-week, 3×/week walking exercise training program would alter corticospinal excitability among people with walking disability due to progressive MS. We report four main findings. First, exercise training resulted in short-term enhancement of corticospinal excitability in both brain hemispheres, which was lost when reassessed during follow-up 3 months later. Second, participants' intracortical inhibition was decreased after training; however, this effect was also short term (lost at follow-up) and was restricted to the hemisphere corresponding to the stronger hand. Third, the training augmented lean mass and reduced body fat, and although there was no change in cardiorespiratory fitness measured as peak of oxygen consumption, capacity to perform exercise (workload) was increased after training and sustained at follow-up (51). Finally, enhancement in corticospinal excitability in the hemisphere corresponding to the stronger hand was correlated with reductions in both severity and impact of fatigue on everyday life (FSS and MFIS, respectively).

### Physical Exercise Training to Enhance Corticospinal Excitation in Progressive MS

Motor thresholds and MEP amplitudes are considered indicators of corticospinal excitation, mediated by glutamate and its activity on *N*-methyl-d-aspartate (NMDA) and α-amino-3-hydroxy-5-methyl-4-isoxazolepropionic acid (AMPA) receptors (24, 30). In fact, higher glutamatergic receptor activity is associated with greater capacity for synaptic plasticity (72, 73), and disruption of this excitatory circuitry is responsible for diminished neuroplasticity and lower capacity to learn new tasks and recover from neurological damage (e.g., aging, stroke, MS) (4, 7, 71). Therefore, there are important initiatives underway to develop new treatments (e.g., exercise, pharmacological, non-invasive brain stimulation) aimed at increasing glutamatergic-mediated brain excitation in the injured brain to enhance neuroplasticity and recover function (9, 45, 71, 74–76). For instance, studies using TMS have confirmed that, in comparison to those who are less physically active, individuals with higher fitness have lower motor thresholds and higher MEP amplitudes (29) (i.e., higher corticospinal excitability) and demonstrate superior increases in MEP amplitudes (i.e., greater neuroplastic response) following paired associative stimulation to induce neuroplasticity (28, 77).

We have previously shown that acute exercise increases corticospinal excitation (i.e., higher MEP amplitude) and reduces intracortical inhibition (i.e., shorter CSP) among people with walking disability due to progressive MS (47). Importantly, this effect was noted only in the stronger hand (47), likely due to a more intact (i.e., less affected) contralateral corticospinal representation (33). Here, we showed bilateral reductions in AMT, increases in MEP amplitudes, and superior motor neuronal recruitment (higher recruitment curve slope) after 10 weeks of aerobic exercise training. This suggests that the stimulus from regular exercise training may have led to the chronic enhancements in excitatory synaptic transmission noted in these participants. Moreover, even though the hemisphere corresponding to the weaker hand, which was likely more affected by MS (33, 78), was unresponsive after one exercise session (47), in this longer term exercise training, it demonstrated capacity to improve in synaptic excitatory transmission. It is interesting to observe that Nicoletti et al. (9) recently reported enhanced corticospinal excitation in people with progressive MS after 4 weeks of d-aspartate treatment, which aimed to enhance NMDA receptor activity (9). They also showed increases in MEP amplitudes following intermittent theta burst stimulation (i.e., enhanced neuroplasticity) (9). It appears that exercise training has comparable benefits in terms of enhancing capacity for neuroplasticity in progressive MS. It is important to note that the corticospinal excitability enhancements reported here and those by Nicoletti et al. (9) were short term and disappeared 3 months after cessation of the intervention. Therefore, we suggest that treatments that enhance neuroplasticity, such as physical exercise training, should be prescribed continuously in progressive MS to protect the brain, improve brain function, and likely to potentiate the effects of treatments (e.g., drugs) and other neuroplasticity-inducing protocols (e.g., non-invasive brain stimulation).

### Physical Exercise Training to Reduce Intracortical Inhibition in Progressive MS

When applying suprathreshold TMS stimulations to the primary motor cortex with participants performing a tonic muscle contraction of the contralateral target muscle, the length of the period of cessation of muscle activity (CSP) is an indicator of intracortical inhibition mediated by the activity of the inhibitory neurotransmitter GABA on its ionotropic and metabotropic receptors (GABA_A_ and GABA_B_, respectively) (24, 32). Although the cortical and spinal contribution to the CSP length is still unclear (24, 79), it is generally accepted that the cortex is the main modulator of CSP change (32). Because excessive GABAergic-mediated intracortical inhibition is considered pathological (80, 81), detrimental to neuroplasticity (39, 40, 81, 82), and is associated with disease progression in MS (36) and stroke (83), decreasing its activity is an attractive treatment strategy to boost neuroplasticity (40, 81).

In healthy people and people with stroke, studies have confirmed that even a single bout of aerobic exercise is able to acutely reduce short intracortical inhibition (59, 83–86) assessed with TMS paired pulse, a TMS biomarker of GABA_A_-receptor activity (24). We recently reported a similar effect after acute aerobic exercise in people with progressive MS (47). Interestingly, here, we showed that after 10 weeks of exercise training, CSP duration was reduced at all TMS intensities, indicating reductions in both GABA_A_ and GABA_B_-mediated intracortical inhibition. This result aligns with findings in healthy individuals demonstrating that 4–12 weeks of strength exercise training reduced both GABA_A_- and GABA_B_-receptor activity, as decreasing in short-intracortical inhibition and duration of the CSP elicited at higher TMS intensities, respectively (26). We have previously shown that among people with MS, superior cardiorespiratory fitness was related to shorter CSP (44). In our present findings, although there were no significant improvements in cardiorespiratory fitness measured as the peak of oxygen consumption (VO_2peak_), there were other indicators of improved physical health (48) such as higher capacity to perform exercise (i.e., kcal/session), greater lean mass, and lower body fat percentage, and increases in other parameters of cardiorespiratory fitness such as the oxygen uptake efficiency slope [for details, see (51)]. The fact that the beneficial reduction (acute and long term) in intracortical inhibition was only observed in the brain hemisphere corresponding to the stronger hand may suggest a greater neuroplastic potential of inhibitory mechanisms in the hemisphere thought to be less affected by MS. Furthermore, our walking training provided a high degree of task-specific training (18, 87, 88). Ziemann et al. has shown that less GABAergic-mediated intracortical inhibition, assessed with TMS, was essential for motor learning processes from task-specific training to occur (89). Decreasing GABAergic-mediated intracortical inhibition has also been proposed to be an important factor initiating increases in muscular strength (26, 27, 31). Although we did not measure muscular strength (e.g., MVC pre–posttraining), we did note increases in lean mass at post and follow-up as well as improvements in walking function [e.g., walking speed; see (51)]. Altogether, this indicates that long-term physical exercise that utilizes task-specific training in highly disabled people with progressive MS reduces intracortical inhibition and possibly improves and restores physical function through enhanced neuroplasticity. Although, because no correlation between changes in intracortical inhibition, body composition, and walking function was noted, it remains to be answered whether decreasing intracortical inhibition would lead to improvements in learning and restoration of function in people with MS. Future research should examine whether such effects would take place in a larger sample with different walking abilities using a randomized controlled design. As well, because we measured overall gains in walking function (51) and body composition, future research should examine whether the enhanced plasticity (reduced inhibition) measured in the hemisphere corresponding to the stronger side of the body indeed translates into global brain function improvement (60) (e.g., bilateral and cognitive function) or whether it is restricted to the contralateral representation. This would be an important discovery for interventions aiming at improving function of the most affected side.

It is interesting that, when compared to healthy controls, some studies have shown reduced intracortical inhibition (shorter CSP) in MS patients (90, 91). Nantes et al. reported that shorter CSP correlated with lower whole brain cortical volume (MRI, magnetic transfer ratio) in progressive MS and that, interestingly, longer CSP was a predictor of upper extremity motor dysfunction (92). Therefore, when compared to the healthy central nervous system (CNS), the CNS affected by MS may display decreased activity of inhibitory mechanisms that, curiously, may work as a compensatory mechanism during brain disease. The concept that there are compensatory mechanisms that increase brain excitation and decrease brain inhibition in order to preserve brain function in CNS disease has been recently proposed by other authors (7, 33, 44, 93–95). However, these processes are certainly not uniform across CNS disorders. For instance, in Parkinson's disease, Fisher et al. (96) showed that high-intensity treadmill exercise program improved walking performance and lengthened CSP time (96), which is typically shortened in people with Parkinson's disease (97). Thomas et al. (98) also showed lengthening of CSP in people with incomplete spinal cord injury after a regimen of treadmill training. Although the mechanisms are not entirely clear, our work and the work of others suggests that rehabilitation and exercise prime the CNS as measured by shifting of the CSP.

### Corticospinal Excitability and Fatigue in MS

Fatigue is one of the most disabling symptoms in MS (44–46). Although the etiology of MS-related fatigue is not completely understood, neuroimaging studies [e.g., MRI, functional MRI (fMRI)] have proposed that its development and progression is due to structural and functional abnormalities in both cortical and subcortical areas (45). Previous studies have shown that 10–12 weeks of physical exercise training can lessen subjective fatigue in people with MS (99), including progressive MS (51, 100). Based on previous findings showing an association between shorter CSP and lowered levels of subjective fatigue in a cohort of people with MS (44), we proposed that improving fitness through exercise training could mitigate fatigue by decreasing GABAergic-mediated intracortical inhibition (i.e., shortening CSP). In this current pilot study, we reported a strong association between increases in corticospinal excitation (recruitment curve; AUC) and reductions in subjective fatigue (FSS and MFIS). Nicoletti et al. (9) also demonstrated reductions in subjective fatigue (FSS) and increases in corticospinal excitation (intracortical facilitation) after d-aspartate treatment in people with progressive MS (9). Furthermore, Créange et al. (101) have also shown increases in corticospinal excitation (e.g., RMT reduction) and reduction in levels of fatigue after erythropoietin treatment to improve walking in people with progressive MS. Our results and the results of others support that there is a link between corticospinal excitation/inhibition and fatigue, which should be examined in larger trials. In fact, non-invasive brain stimulation methods (repetitive TMS, transcranial direct current stimulation), which aim to increase cortical excitation and treat MS fatigue, have been recently proposed (45). It is important to note that the abovementioned experiments, and the present study, measured perceived (i.e., subjective) fatigue and not fatigability (i.e., muscle/performance fatigability measured during contraction). Nonetheless, because perceived fatigue and fatigability closely associate (102), our results showing reduced levels of perceived fatigue and improved fitness suggests that following training, subjects required less physical effort to perform activities of daily living, suggesting superior energy availability and reduced fatigability (102). Therefore, we propose that exercise training might be able to mitigate symptoms of fatigue possibly by acting through increases in excitatory circuitry.

### Limitations

There are some important limitations to consider when interpreting the results of the present study. First, this was a small pilot study, and no statistical sample size calculation was conducted for the outcomes presented in this manuscript, which limits the statistical power to obtain conclusive results. Second, no control group was included, which limits the conclusion on the true effect of the intervention. Third, as only patients with progressive MS and severe MS-related walking disabilities (EDSS, 6.0–6.5) were included, the findings may not be applicable for relapsing–remitting and/or less disabled MS patients. Despite these limitations, the novel insights from this study may serve as a rationale for larger studies and continued efforts in investigating the effects of exercise and physical rehabilitation on neuroplasticity and functional recovery in MS.

As for considerations for future studies, although the aim of this study was to investigate changes in corticospinal excitability in a non-exercised hand muscle to demonstrate widespread effects of exercise training on global brain plasticity (59, 60), investigating muscles that were more involved in the walking training (e.g., lower limb muscles) could provide more insight regarding the link between the trained muscle and cortical function (TMS) (27). Moreover, having participants' neuroimaging data (e.g., magnetic resonance imaging) could help to better understand the role of lesion volume and location on exercise-induced corticospinal excitability changes. We determined averaged MEP amplitudes and CSP times from a small number of trials (three to six) as done previously by others (28, 65, 66), and with participants performing tonic contraction, in order to reduce intrasubject variability (27). Future studies should examine the optimal number of stimulation trials (103) in order to produce reliable MEP/CSP data. With respect to the TMS recruitment curve parameters, we used linear regression (TMS intensities by MEP amplitudes), as done by others (25, 34), in an attempt to assess the corticospinal tract recruitment gain (slope) and accuracy (*R*^2^); biomarkers were previously proposed by Potter-Baker et al. (67) to reflect morpho-physiological integrity of the corticospinal tract in stroke. However, more studies are necessary in order to understand what the best model is [e.g., sigmoidal (67) or linear (25, 34)] when calculating these parameters while taking into consideration the different TMS methodologies (e.g., range of TMS intensities employed), the clinical population (e.g., stroke, MS), and lesion profile (e.g., lesion volume, location).

## Conclusion

To our knowledge, this is the first study to investigate longer term effects of exercise on corticospinal function using TMS in patients with progressive MS. This exploratory pilot study provides evidence that a neuroplastic potential still exists in patients with progressive MS and severe MS-related walking disability. Specifically, we found that 10 weeks of vigorous treadmill training reduced intracortical inhibition and increased corticospinal excitability. These corticospinal adaptations were predominately found in the brain hemisphere corresponding to the stronger hand, suggesting a greater neuroplastic potential in the hemisphere that may be less affected by MS. Moreover, the exercise-induced enhancement in cortical excitation was associated with reductions in fatigue, suggesting this as a potential mechanism involved in the effects of exercise on fatigue. The novel findings from this pilot study highlight the importance of long-term exercise efforts—even in patients with progressive MS—and can serve as a rationale for future studies and continued efforts in investigating the effects of exercise on the brain.

## Data Availability Statement

The data supporting this study are available at request from the corresponding author at the Memorial University of Newfoundland, Canada.

## Ethics Statement

The studies involving human participants were reviewed and approved by Health Research Ethics Board. The patients/participants provided their written informed consent to participate in this study.

## Author Contributions

AC, AD, and MP: conception or design of the research. AC, RP, and AD: data collection. AC and AD: data cleaning and analysis. AC, MR, and MP: writing and editing the manuscript. All authors interpretation of data, final approval and revision of the version to be published, and agreement to be accountable for all aspects of the work.

## Conflict of Interest

The authors declare that the research was conducted in the absence of any commercial or financial relationships that could be construed as a potential conflict of interest.
